# Cohort Profile: the SMRU Refugee and Migrant Pregnancy Study in Western Thailand and Eastern Myanmar

**DOI:** 10.12688/wellcomeopenres.25582.1

**Published:** 2026-01-07

**Authors:** Rose McGready, Nicholas J White, François H Nosten, SMRU Refugee and Migrant Pregnancy Study Collaborators

**Affiliations:** 1 Mahidol-Oxford Tropical Medicine Research Unit, Faculty of Tropical Medicine, Mahidol University, Shoklo Malaria Research Unit, Mae Ramat, Tak, 63140, Thailand; 2 Centre for Tropical Medicine and Global Health, University of Oxford Centre for Tropical Medicine and Global Health, Oxford, England, UK; 3 Faculty of Tropical Medicine, Mahidol University, Mahidol-Oxford Tropical Medicine Research Unit, Mae Ramat, Tak, 63140, Thailand

**Keywords:** Anaemia, Malaria, Maternal mortality, Migrants, Neonatal mortality, Non-communicable diseases, Nutrition transition, Pregnancy, Refugees, Stillbirth

## Abstract

**Background:**

Marginalised populations face significant health risks in pregnancy with reduced access to preventive and life-saving services due to conflict and migration. Infectious disease risk is high and the double burden of malnutrition increases risk from non-communicable disease although only weak epidemiological data supports this in refugees and migrant communities. This manuscript describes the SMRU Refugee and Migrant Pregnancy Cohort commencing nearly 40 years ago, established in response to the very high rate of *Plasmodium falciparum* maternal mortality in refugee camps on the Thailand Myanmar border

**Methods:**

Pregnant women who registered to antenatal care clinics of the Shoklo Malaria Research Unit from 1986 to 2024 living in marginalised communities of refugee and migrants were the eligible population. Pregnancies were prospectively followed from enrolment through to childbirth. Types of data include: 1) medical and obstetric records including patient characteristics, pregnancy progress and birth outcomes and 2) investigations (such as HIV).

**Results:**

Among 94,645 pregnancies maternal mortality was 176 per 100,000 livebirths (120/68,024). Embedded cohorts included observational and clinical trials, providing evidence on the optimisation of treatment of malaria in pregnancy and on the rapid changes towards non-communicable diseases in refugees and migrants. Low mean height (151.4 cm), well below European and American populations from which the majority of guidelines have been created, questions appropriateness, such as gestational weight gain in pregnancy. A broad scope of research findings including tropical infections impacting pregnancy outcomes, mental health and suicide, a shared platform of “-omics” of Karen and Burmese women from first trimester, and practice of care in low-income settings have emerged and been shared.

**Conclusions:**

The SMRU Refugee and Migrant Pregnancy Cohort findings have had significant local and international impact including changing the World Health Organisation Malaria Treatment Guidelines in pregnancy; and establishing a range of guidelines and tools improving maternal-child health practices.

## Plain language summary

**Cohort Profile in a nutshell**

- The SMRU Refugee and Migrant Pregnancy Cohort is a population-based study including nearly 100,000 women, enrolled from 1986 to 2024. It includes all eligible women.
- Data typical of pregnancy records globally has been collected and the progress of pregnancy (weight, fundal height, fetal position, blood pressure, haematocrit) and pregnancy outcomes have been recorded by trained staff with labour monitored using the modified WHO partograph.
- After nearly 40 years of data collection 94,645 refugee and migrant women registered and: more than 6 in 10 had ultrasound confirmation of gestation, just over 1 in 10 women had malaria in pregnancy; and of known outcomes 13 in 1,000 newborns were stillborn and 1.8 in 1000 mothers died in relation to pregnancy.
- SMRU Refugee and Migrant Pregnancy Cohort uses a data on reasonable request model.

## Why was the cohort set up?

In 1985 *Plasmodium falciparum* malaria in pregnancy alone was responsible for a staggering estimated maternal mortality of 1,000 per 100,000 live births in refugee camps on the western border of Thailand.

The problem of drug-resistant strains of *P. falciparum* affecting the Greater Mekong Subregion was in part conflict driven: the Vietnam War (1955–1975), the Khmer Rouge siege of Cambodia (1975–1979) and prolonged political instability in Myanmar (1948 to present) ^1^. Conflict is associated with mass population displacement alongside disruption of health care and in 1986 the centuries old drug for treatment of malaria, quinine, was only effective for uncomplicated malaria in combination with other medications in South East Asia, and the mainstay drug for prevention in pregnancy sulphadoxine-pyrimethamine (SP) was already lost to resistance ^2^.

**Figure 1.**
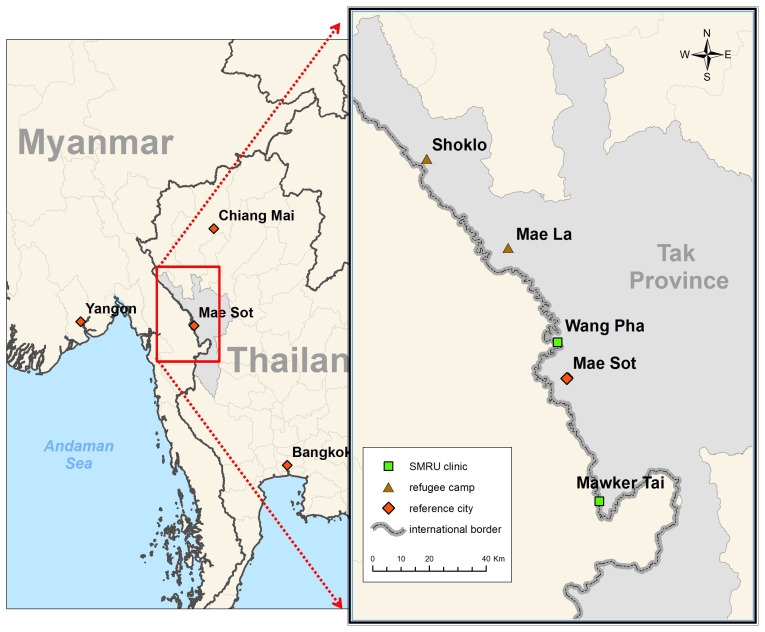
Two main refugee camps on the Thailand-Myanmar border where SMRU has worked 1986–2024.

Médecins Sans Frontières (MSF) was one of the first non-government organisations (NGOs) to respond to the tens of thousands of predominantly Karen and Karenni refugees crossing into Thailand along with Committee for Coordination of Services to Displaced Persons in Thailand (CCSDPT). From 1984 multiple temporary shelters were established in forested areas (with local malaria transmission) including Shoklo Refugee camp and the evolution of the camps over time has been documented by The Border Consortium ^3^. The two main camps SMRU has worked on maternal and child health (MCH) are Shoklo and Maela ( Figure 1).

With MSF documenting falciparum malaria as the major cause for death and hospitalisation in the camps one of their doctors (FN) at the time, urgently consulted a malaria expert (NJW) and together they formed a partnership to address the problem. At the time there was a paucity of information available on the effects of malaria in pregnancy and on the impact of drugs used for treatment on the fetus, in areas where immunity to the infection was low or non-existent ^4^. A doctor with obstetric skills (RM) further supported the SMRU Refugee and Migrant Pregnancy Cohort Study and maintained the objective of reigning in preventable malaria related, and all cause maternal deaths.

In 1986 in another part of the world, DJ Barker and C Osmond published the first of a host of geographic disease analysis (England and Wales), where they linked poor nutrition in early life to increased susceptibility and higher mortality from ischaemic heart disease in adulthood ^5^. The impact of the global epidemiological transition from infectious diseases to nutrition related chronic diseases on maternal and fetal health remains poorly understood in highly marginalized communities, largely due to the difficulty of conducting longitudinal studies in such settings ^6, 7^. The SMRU Refugee and Migrant Pregnancy Cohort Study bears witness to this ongoing health transition within a vulnerable population with longitudinal cohort data collected since 1986. It was never envisaged that the study would be enrolling women four decades later yet it offers an opportunity to investigate trends in maternal and newborn health and to inform strategies aimed at reducing health inequalities in this unique population ^8^.

## Methods

### Who is in the cohort?

There are nearly 100,000 women (end 2024) in the SMRU Refugee and Migrant Pregnancy Cohort Study aged 13–53 years. Entry has been open and non-selective with the only requirement being evidence of pregnancy and registration at SMRU clinics, and in the early days, including the MSF antenatal clinic. Registrations commenced in 1986 in refugees and in 1997 in migrants. SMRU left the camps at the end of 2016 although the recent collapse of USAID supported health services has led to re-engagement in Maela refugee camp. Migrant enrolments continue through to the present day (time of writing).

SMRU is a field station of the faculty of Tropical Medicine, Mahidol University, Bangkok, Thailand and part of Mahidol Oxford Research Unit (MORU), which was located in Mae Sot city, Tak Province, north-western Thai-Myanmar border until 2022 and then moved to Mae Ramat district. SMRU has had a dual role of conducting research activities and providing humanitarian services to the population with a focus on infectious diseases, such as malaria and tuberculosis, and on maternal and child health (MCH).

***Refugee population***. Thailand is not an official signatory to the 1951 United Nations (UN) Refugee Convention, preferring to use the term “temporary shelters” however United Nations High Commission for Refugees ^9^ operates in Thailand on a cooperative basis and refugees are still registered under UNHCR processes. Refugees started arriving in Thailand in the mid 1980’s. Médecins Sans Frontières (MSF) arrived in 1984 and SMRU was established in 1986 in Shoklo camp, with collaborative efforts to reduce mortality particularly in pregnant women and young children ^10, 11^. In 1995 border skirmishes inside Thailand resulted in the Thai Ministry of Interior amalgamating the displaced peoples’ camps into the nine camps present today, sheltering approximately 91,000 refugees (December 2024) ^3^. The total camp population in 2024 is almost identical to that in 1995, though many fluctuations have occurred over the years. Between 2006 and 2017, a total of 109,402 refugees from the camps were resettled in third countries supported by UNHCR. Although a programme for facilitating voluntary repatriation was agreed between the Thai and Myanmar governments in 2016, fewer than 1,000 refugees returned to Myanmar. Plans to return collapsed with the Rohingya genocide on Myanmar’s western border starting in 2016.

Shoklo camp was the final camp merged into Maela camp in 1998 making it the largest of all the camps, although SMRU moved in 1996 ( Figure 1). The first ANC was held on a mat under a piece of plastic to protect from the sun, finding back 16 women who were already registered with SMRU from other camps. Word of mouth connected people and messaging through camp leaders also prevented loss to follow-up. In Maela camp, MSF handed over to Aide Médicale Internationale (PU-AMI) in 2005, and PU-AMI to the American Refugee Council, and shortly thereafter to the International Rescue Committee (IRC) in 2015. SMRU handed over maternal and child health in refugees at the end of 2016 to IRC due to increased health service needs in the expanding migrant population.

***Migrant population***. It is estimated that there were 2.3 million Myanmar migrant workers in Thailand in 2023 and Tak Province is home to an estimated 200,000 of these workers although 2024 estimates suggest this has increased ^12^. Migrants with work permits have access to healthcare in Thailand while undocumented migrants have to pay (debt incurring, catastrophic costs) provided they can navigate the language. Complex and changing document requirements have resulted in a greater population of undocumented than documented migrants along the border. Furthermore, 40–45% of migrants in Thailand are women, and safe motherhood programmes are required but scare ^12^. Most undocumented migrants in rural Tak Province work in the agriculture sector and depend on daily wages. In 2017 Dreamlopments introduced The Migrant Fund or M-FUND ( https://www.dreamlopments.com/) a low cost health insurance package available regardless of documentation status ^13^.

**Figure 2.**
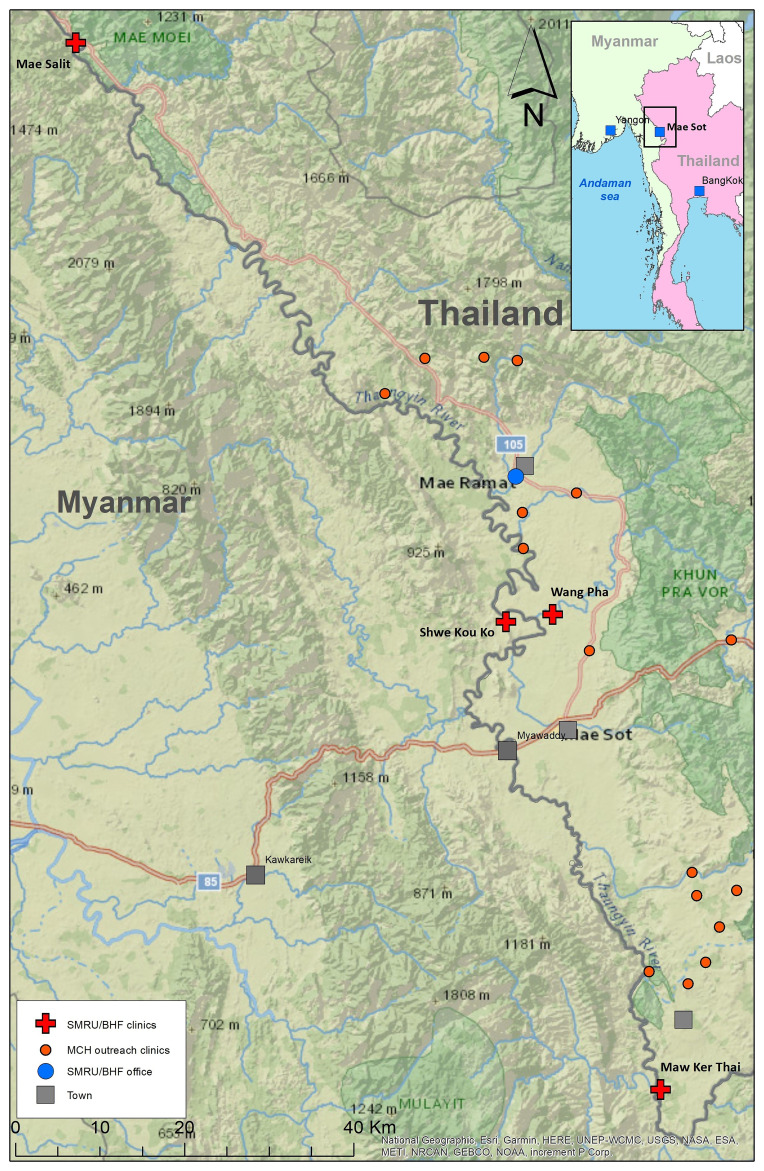
SMRU clinics: fixed (red cross) and outreach (red circles), Thailand Myanmar border.

SMRU commenced outpatient and in-patient department services to undocumented migrants in 1996 and established ANC clinics in 1997. For more than two decades, two fixed clinics just inside the western border of Thailand; Mawker Thai (MKT) and Wang Pha (WPA), have provided outpatient, inpatient and ANC services for migrants. Two clinics providing only mother and child health services in Myanmar opened in 2017 at Mae Salit (MSL) and Koko (KK) in 2020. Multiple outreach clinics opened in 2021 ( Figure 2). Birth rooms opened in December 2007 in WPA, April 2010 in MKT, January 2017 in MSL and March 2020 in KK.

### How often have they been followed up?

Malaria dictated the frequency of ANC visits at SMRU. The malaria-related maternal mortality ratio was estimated at 1,000 per 100,000 live births before the introduction of weekly ANC visits in 1985–6 ^11, 14^. The rationale for the frequent visit policy adopted at SMRU was based on the life cycle of the *P. falciparum* parasite, which has an incubation period of 11–15 days ^15^, and SMRU has observed maternal deaths from malaria with women being absent from ANC for as little as one week ^14^.

The SMRU strategy differed from the World Health Organisation (WHO) recommendations for control of malaria in pregnancy. The mainstay of the WHO strategy since 2001 has been intermittent preventive treatment with sulphadoxine-pyrimethamine from 16 weeks gestation (3 doses at least one month apart), provided with bed nets and screening for malaria in symptomatic women (or passive case management). High levels of drug resistance to sulphadoxine-pyrimethamine precluded its use and insecticide treated bednets were not found to be significantly protective against *P. falciparum* parasitaemia in pregnancy ^16^. With maternal mortality at 1% from passive case management the only viable alternative at the time was active case finding i.e. frequent regular screening and treatment for any positive, regardless of symptoms, across gestation. This was recommended for NGOs border wide and was one of the first published findings of the cohort ^11, 14^. As malaria infection control in the non-pregnant population improved in refugee camps with deployment of artemisinin-based combination therapies (ACTs) the frequency of ANC visits was decreased; the initial once weekly malaria screening was revised to once every two weeks (2008), and then to the current policy, the first three ANC visits and thereafter only if indicated (fever, history of fever or symptoms of malaria headache, body, muscle or joint pain, dizziness). However, women diagnosed with malaria infection were also screened as often as possible, every ANC visit until delivery due to the risk of further episodes from drug failure, recurrence of *P. vivax* or another infectious bite ^17^.

### What has been measured?

SMRU Refugee and Migrant Pregnancy Cohort Study core variables are summarised ( Table 1) and have been consistently collected over time, although collections of some variables were initiated as the cohort aged and adapted.

**Table 1.** Core data collected in the SMRU Refugee and Migrant Pregnancy Cohort Study.

Phase	Measurement (year introduced)
Baseline	Age, ethnicity, gravidity, parity, number of living children, number of abortions (miscarriage), literacy, smoking (1996), maternal weight, height and MUAC (2004), Obstetric History, estimated gestational age by ultrasound (since 2001), Screen for HIV (2001–2006 opt in, 2007 universal), Syphilis (2008), Hepatitis B (2008)
Routine screening during pregnancy	Malaria screen (positive or negative), and if positive additional variables were collected for each episode: date, EGA, antimalarial treatment; plasmodium species; parasitaemia, symptoms (fever and history of fever in the past 48 hours). Anaemia screen, blood pressure, symphysis fundal height (and ultrasound for growth and fetal wellbeing if indicated), fetal position, fetal heart rate, temperature, maternal weight, screening for gestational diabetes (risk factor based OGTT from 2011, universal two step GCT then OGTT 2021).
Morbidity during pregnancy, delivery and post-partum	All morbid events e.g. non-malaria fever, malaria, non-communicable disease diagnosis including gestational age at event, diagnosis and treatment, and investigations e.g. fever urine and blood culture, CBC, CRP (as a minimum from 2010)
Labour	Partogram (1994) to monitor the progression of labour (cervical dilatation, fetal head decent, uterine contraction), detect complications (Obstructed labour, meconium-stained liquor, fetal heart rate abnormalities) and perform early interventions (Augmentation of labour or transfer for Caesarean delivery) in order to improve the maternal and neonatal outcomes.
Delivery mother	Mode of delivery, estimated blood loss in ml by weight (2009), mother alive or died, placental weight
Neonate	Live or stillborn, sex, birth date, birth weight (date, time), length, arm circumference, Apgars 1, 5 minutes, resuscitation, newborn examination
Infant follow-up	Neonatal survival, admission to special care baby unit (SCBU from 2008). Infant mortality in specific sub-cohorts.

Abbreviations: CBC complete blood count; CRP C-reactive protein; EGA estimated gestational age; HIV human immunodeficiency virus; GCT glucose tolerance test; ml milliliters; MUAC mid upper arm circumference; OGTT oral glucose tolerance test; SCBU special care baby unit.

Medical events and their treatment and all prophylaxis with micronutrient supplementation for the entire pregnancy are individually recorded. Treatment guidelines systematised common infection management.

At every ANC visit, maternal weight, blood pressure, symphysis fundal height, fetal position and fetal heart rate were routinely measured. Associate Prof Lilly Dubowitz came frequently to Shoklo camp in 1993–94 and later to Maela to teach and ensure quality control of the Dubowitz gestational age assessment and later neurological assessment in newborns and infants ^18^. Gestational age at first ANC has been by ultrasound since late 2001. Statisticians supported work on multiple symphysis fundal height measurements compared to ultrasound to assign gestational age when LMP was not known; and further utilised to identify the fetus at risk of intrauterine growth restriction at routine ANC visits ^19^. Modified WHO partographs have measured labor progress from 1994 and high-risk pregnancies in labour are managed according to the SMRU obstetric manual, with referral if required. On either side of the border, women who require Caesarean delivery for obstetric indications, such as placental praevia or cephalo-pelvic disproportion, are transferred to the nearest hospital in the emergency standby SMRU car including 5–10% of women over the years.

The complications of newborn babies such as neonatal jaundice, neonatal sepsis, transient tachypnoea of the newborn and feeding difficulties are managed following SMRU neonatal guidelines in a special care baby unit (SCBU): established in Maela refugee camp in 2008, in Wang Pha clinic in 2009, in Maw Ker Thai in 2010, in MSL in 2017 and in KK in 2020. Follow-up beyond birth was conducted differently in the camp and migrant settings. In the camps infants had a visit at one month or the home visitor went to find them if they did not show up. This standard was not possible routinely in the dispersed migrant communities but some cohorts followed infants after birth including vaccination cohorts.

SMRU has supported provision of contraceptives including short- and long-term methods, available at both fixed and outreach clinics ^20, 21^ and at times, tubal ligation services ^22^.

Over the decades SMRU has ensured quality assurance through standardized curriculums, refresher training, internal quality control conducted by the SMRU Training Department and standardised protocols contained in guidelines. These guidelines include the SMRU malaria treatment guidelines, Burmese Border Guidelines (BBG) for general medicine and paediatrics, SMRU Obstetric Manual and the SMRU Neonatal Guidelines. Examples of the quality of health care delivered by the staff (medic, nurse, midwife) has been assessed regularly ^23– 25^. Emergency obstetric and newborn care has been implemented using the Advanced Life Support in Obstetrics (ALSO ^®^) course developed by the American Academy of Family Physicians. Good clinical practice (GCP) training for research, including face-to-face and online training, has been provided to all SMRU staff and renewed as necessary.

### Data source and who collected the data?

The first antenatal care (ANC) record was the MSF card in French and English, a single A4 two-sided card. The card was used in all camps and collected when the women gave birth, had a miscarriage or left the study area; with local Karen staff trained to complete the records in English (notations in Karen and Burmese were translated). Manual extraction of information from the cards was entered into a computer-based record using the database management system (DBMS) dBase Database File (DBF) the standard desktop database of 1980s and 1990s. Minor changes were made to the ANC card to improve clarity of data collection in 1998 and this was entered prospectively, once per week. In 2007 an improved design, similar to a study Case Report Form (CRF) was introduced. In late 2009 a major upgrade was undertaken and supported by a non-profit organization called Technologies Sans Frontières. Working alongside the SMRU Information Technology team and obstetric doctors a unique computer-based platform for individual participant records was modelled off the CRF. Staff were trained in how to use the computer to enter the data in real time. This took 18 months to have all sites competent, continuing the old system while practicing the new. Data verification is optimized by flagging unusual vales. It is reliant on the data entry person but subjected to checks by different staff in different areas of the clinic as a woman moves through different units to complete her antenatal care visit. The locally trained medical staff and doctors review all records before a woman departs from the clinic each day. Data changes can be tracked. Women still receive a hand-held pregnancy record (in English, Burmese and Thai) so wherever they end up in medical care (Myanmar, Thailand or other clinic) their information can be used to provide appropriate care.

### Ethics

Approval from the Ethical Committee is obtained (Ethics reference: TMEC 17–027; OxTREC 583–16). For sub-cohort analysis separate ethical approval and individual informed consent was sought and this has been included in the methods section of each manuscript ( Table 2).

**Table 2.** Research publications from the SMRU Refugee and Migrant Pregnancy Cohort Study.

Subject		Reference
Malaria	Treatment of malaria in pregnancy	17, 28– 56
	Antimalarial pharmacokinetics-pregnancy & lactation	57– 77
	Prevention of Malaria in pregnancy	78– 82
	Epidemiology of Malaria in pregnancy, infants	11, 14, 83– 93
	Placental malaria	94– 100
	Antibodies and malaria and pregnancy	101– 109
Other infections	Other infections e.g. Aeromonas spp. COVID-19, Hepatitis-B, HIV, pyelonephritis, scabies, syphilis, soil transmitted helminths, toxoplasmosis, typhus	23, 110– 122
Obstetrics LIC	Castor oil induction, Contraception, Distance, Folate, Gestational age assessment, Gestational Trophoblastic Disease, Ketamine T/L, Nuchal cord, Safe Motherhood, Twins	19– 21, 24, 123– 131
Neuro-development	Neurodevelopmental - limited resource settings	18, 132– 134
NCD	Hypertensive disorders of pregnancy, gestational diabetes	135– 137
Nutrition	Micronutrients in blood, breast milk, gestational weight gain	138– 146
	Thiamine (Vitamin B1) deficiency in Refugees	147– 149
	Smoking, betel nut and alcohol use in pregnancy	150– 152
Adolescent	Adolescent pregnancy	153– 155
Literacy	Low health literacy	156, 157
Anaemia	Anaemia and blood disorders	158– 161
Ethics	Research Ethics in pregnancy	162, 163
Perceptions	Perceptions of care	164– 166
Training	Teaching and learning including ALSO ^®^	167– 170
Sub-Cohorts	ARI – Acute Respiratory Infection in 1st year of life, neonatal care	25, 171– 176
	NUT – Asia Mix MMN Refugee Camps study	177– 179
	UPS – Ultrasound Pregnancy Study	180– 186
	SUC – Susceptibility Post-partum malaria	187– 190
	NJS – Neonatal Jaundice Study and G6PD deficiency	191– 201
	MHS – Mental Health Study	202– 212
	Hepatitis-B and TDF for prevention MTCT	213– 222
	MSP – Molecular Signature Pregnancy	223– 232
	Interbio-21 Study	233– 240

HIV human immunodeficiency virus, LIC low-income countries, NCD – non-communicable disease, T/L tubal ligation

### Public and patient involvement

This cohort commenced with medical staff from MSF and SMRU living in the camps with the refugees at the Thailand Myanmar border, witnessing the high mortality rates from malaria, with some infected with malaria themselves. Trained health care workers were from the camps. In 2008, increased community engagement resulted from the Tak Province Community Advisory Board in Thailand. This group, comprised of community members were asked to advise design, process and outcomes of interest, and subsequently approved (or disapproved) all studies conducted at SMRU ^26, 27^.

## Results

### What has it found?

**Figure 3.**
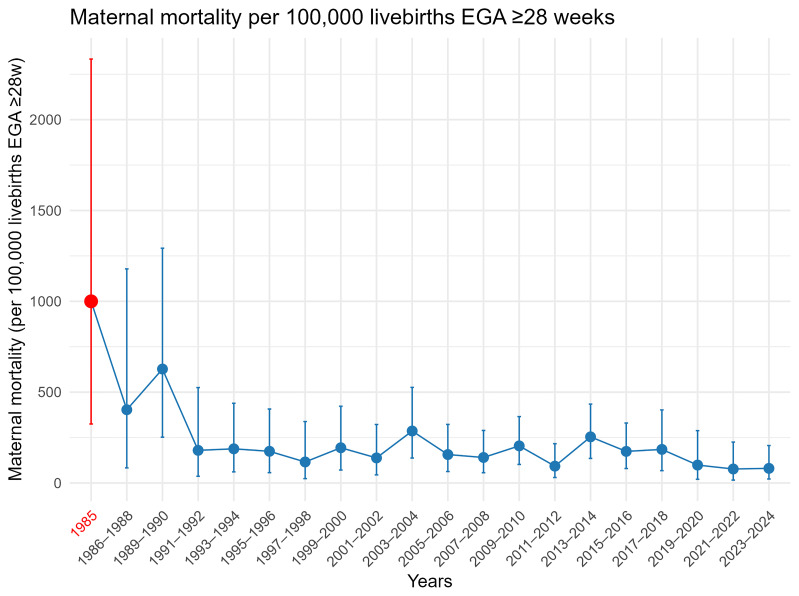
Maternal mortality ratio per 100,000 livebirths(95%CI), in 1985 [red] due to malaria, 1986–2024 [blue] all-causes.

After nearly 40 years 94,645 refugee and migrant women registered: 11.7% (11,100/94,6438) had malaria in pregnancy (7 not tested), 0.9% (833/94,645) were multiple pregnancies, 66.0% (62,496/94,645) had ultrasound confirmation of gestational age (1.7% (1,605/94,645) had missing gestational age). After excluding 18.6% (17,600/94,645) women lost to follow-up before pregnancy outcome was known and/or multiple pregnancies, 10.0% (7,617/76,252) were miscarriage, 90.0% (68,635/76,252) birthed singletons. Stillbirth occurred in 1.3% (862/68,185) of newborn outcomes (at 24 weeks or more gestation, 141 outcomes missing). Maternal mortality ratio was 176 per 100,000 livebirths (120/68,024) livebirths of singleton or 1 ^st^ born twin, of 24 weeks or more gestation). Two main highlights from the SMRU Refugee and Migrant Pregnancy Cohort Study include:

i) The very high risk of *P. falciparum* malaria related maternal mortality in areas of low transmission ^14^ and the stalled maternal mortality rates for two decades ( Figure 3).

Beyond maternal mortality, the cohort has demonstrated the strong association between malaria (both *P. falciparum* and *P. vivax*) during pregnancy and severe adverse pregnancy outcomes such as maternal anaemia, foetal loss including miscarriage ^241^, stillbirth and neonatal mortality ^83^, and poor infant outcomes demonstrating small for gestational age and preterm births as the main mediators of neonatal deaths ^83, 84^. These adverse outcomes occur despite treatment and are observed at submicroscopic parasitaemic concentrations ^242^.

The cohort established significant evidence on treatment which informed WHO and changed its global malaria treatment guidelines to artemisinin-based combination therapies (ACTs) ^29^. ACTS are both more efficacious and safer than previously recommended treatments (like quinine). SMRU conducted multiple randomized controlled treatment trials and was the first study to randomize women to be treated with ACTs in the first trimester, in 2012 ^17^. The malaria in pregnancy evidence included significant clinical and laboratory work including on the pharmacokinetics of antimalarials in pregnancy.

ii) Evidence on the size and scope of the nutrition transition in marginalised populations has been provided by an analysis involving 40 000 women and data spanning 30 years in refugees and 19 years in migrants. Nutritional trends indicate a rapid 1.7-fold increase in high BMI within 13 years; with nearly no change in low BMI (affecting 1 in 7 refugees,1 in 5 migrants) despite significant decreases in infection (malaria), anaemia and smoking. The double burden of nutrition impacts lives that already contend with social, political and economically turbulent times, and this is accompanied by a lack of awareness among health workers, and women with low health literacy, on the ill effects of poor nutrition. In addition, the women in SMRU Refugee and Migrant Pregnancy Cohort who have an average height of 151.4 cm demonstrated why international guidelines e.g. for gestational weight gain, based on taller and heavier populations from North America, Western Europe and Africa are not appropriate ^138^.

Research publications by subject area are provided ( Table 2).

### What are the main strengths and weaknesses of the study?

The main strength of this cohort is the prolonged length of data collection in a rare environment amongst marginalised refugee and migrant women: in contrast to data in refugees and migrants from high-income countries, sites where capacity for data collection is typically more robust than in low resource settings ^243, 244^. Data on refugees and migrants remaining in low resource settings remains pauce and likely sub-optimal compared to countries of resettlement ^245, 246^.

The consistency of the data collected and archiving of records for data queries and clarifications remains another strength of this cohort, remarkable given the fragile state disturbed by intermittent conflict, movements of the camps, natural disasters, influx and efflux of migrants and border closures.

The cohort has been well supported by both basic field laboratories with microscopy for malaria and haematocrit samples from the outset, and later with a malaria in-vitro laboratory established in 1995, a microbiology laboratory in 2006 and a haematology laboratory in 2010 incorporating research of anaemia. Embedded trials have strengthened data collection and improved data richness in some sections of the cohort.

The main weakness of the cohort: women who are lost to follow-up before the outcome of pregnancy is confirmed is also inherent to the instability of the local situation and mobile nature of the population. It is inevitable and important that over the four decades covered by this cohort clinical practices at SMRU have continuously evolved and improved. While the collection of core clinical and demographic variables has been systematically conducted since the early years, certain types of information were introduced only in later periods e.g. testing for gestational diabetes; resulting in systematic missingness. The ethnic makeup of the patient population has also changed over time, from a predominantly Karen refugee population in the early years to a predominantly Burman migrant population in recent years. This shift likely effects genetic and cultural susceptibilities to certain health outcomes e.g. anaemia, however information on ethnic group has always been recorded. Some data such as socioeconomic status is only available in selected cohorts and there is no data on the health of the husband/partner health. In case of referral of the pregnant woman or neonate to the Thai hospital (and less frequently a hospital in Myanmar) certain data was not always available such as Apgar scores.

## Data Availability

Approvals of the study protocol were obtained from relevant local ethics and regulatory frameworks: Ethics Committee of the Faculty of Tropical Medicine at Mahidol University (Ethics reference: TMEC 17–027) and Oxford Tropical Research Ethics Committee (Ethics reference: OxTREC 583–16). Specific sub-cohorts have their own approvals. All data contributing to this study are taken from existing anonymised patient records. Data has been deposited in ORA (27/12/2025). The reserved DOI for this dataset is 10.5287/ora-yjw6d0g9r. The SMRU Refugee and Migrant Pregnancy Cohort welcomes proposals for data access and sharing from bona fide researchers. Enquiries should be directed through the Bioethics and Engagement team coordinates Mahidol Oxford Research Unit’s Data Access Committee ( datasharing@tropmedres.ac). Data has been shared and is accessible with various groups for example: all malaria in pregnancy data with WWARN ^51, 54^, the infant neurodevelopment with Saving Brains program under the Grand Challenges Canada group ^247^; maternal weight and heights with GWG Pooling Project Consortium at Harvard T H Chan ^248^; and the data browsing too for gene and module-level blood transcriptomics as an Oxford library database Database (Oxford). 2024 Apr 2;2024:baae021. doi: 10.1093/database/baae021.
