# Supplementary material for: Cohort Profile: the SMRU Refugee and Migrant Pregnancy Study in Western Thailand and Eastern Myanmar
Source: Wellcome Open Res. 2026 Mar 27;11:21. Originally published 2026 Jan 7. [Version 2] doi: 10.12688/wellcomeopenres.25582.2 (PMC12921400; doi:10.12688/wellcomeopenres.25582.2)
Supplement: Peer review history

# Reviewer report: Reviewer response for version 1

**DOI:** 10.21956/wellcomeopenres.28177.r144721

**Published:** 2026-02-19

**Authors:** Muhammad H Zaman

**Affiliations:** 1 Boston University, Boston, Massachusetts, USA

This is an excellent cohort report, and describes the nature of the cohort, the study, and the results in crisp detail. I learned a lot from this report. I do not have any concerns, but have a few (relatively minor) recommendations.

 1. The notion of migrant and refugee can, at times, be arbitrary in a complex setting. There are people who may fall into both, or neither. How did the researchers deal with various categories and their overlapping nature? and those who did not clearly fit into any group?

 2. Were there community members who were pendular migrants (i.e. moved back and forth between locations)?

 3. How have issues around climate change in the region impacted the study, or its results?  

 4. Finally - as this study developed further and strengthened, over the years, how have the perceptions of access to care, knowledge among the cohort, or practices changed? 

 Overall, this is an extraordinary study and I learned a lot.

Is the study design appropriate for the research question?

Yes

Is the rationale for, and objectives of, the study clearly described?

Yes

Are sufficient details of the methods provided to allow replication by others?

Yes

Are the datasets clearly presented in a useable and accessible format?

Yes

Reviewer Expertise:

Infection in complex settings; Refugee and Migrant Health. See a recent book here:  https://www.press.jhu.edu/books/title/12621/we-wait-miracle?srsltid=AfmBOopky6ty7NKsKWQAK02c6ailGm1c-vS0NcJxsQ6EQQNgD5u2_eic

I confirm that I have read this submission and believe that I have an appropriate level of expertise to confirm that it is of an acceptable scientific standard.

# Response

**Published:** 2026-03-01

**Authors:** Rose McGready

**Affiliations:** Shoklo Malaria Research Unit, Thailand

We sincerely appreciate the useful suggestions from Reviewer 2. Along with the comments from Reviewer 1 the manuscript has improved from the considerable time devoted to review. This is an excellent cohort report, and describes the nature of the cohort, the study, and the results in crisp detail. I learned a lot from this report. I do not have any concerns, but have a few (relatively minor) recommendations.

 1. The notion of migrant and refugee can, at times, be arbitrary in a complex setting. There are people who may fall into both, or neither. How did the researchers deal with various categories and their overlapping nature? and those who did not clearly fit into any group? This is a good point. Refugees and migrants were registered based on where they first attended antenatal care, and in the majority (>99%), women completed care (camp or migrant site) and delivered under the same banner. Documentation of birth for marginalised populations is difficult. Refugees know they need to birth in the camp if they want the child’s birth is registered in the camp…which likely explains why registration and delivery place are usually the same.

 2. Were there community members who were pendular migrants (i.e. moved back and forth between locations)? Few. All pregnant women receive a unique identifier. If they move between locations it is picked up through the coding and it is very infrequent.

 3. How have issues around climate change in the region impacted the study, or its results?   This is an area we are currently working on.

 4. Finally - as this study developed further and strengthened, over the years, how have the perceptions of access to care, knowledge among the cohort, or practices changed? 

   This is a gap/limitation and text to include this has been added prior to the conclusion and referenced (Am. J. Trop. Med. Hyg., 108(3), 2023, pp. 465–469 doi:10.4269/ajtmh.22-0624). Measuring changes in perceptions in pregnancy care has to be captured around the change time and be appropriately timed; at the beginning of change there is frequently resistance e.g. masks for COVID-19. A manuscript currently under review includes perceptions of a new model of ANC care but it was not possible to isolate women who used both models of care. However there have been changes in perception, care and knowledge observed but not captured in publications. For example in the first 10 years of the camp the mortality of infants was 1 in 4 in the first year of life; uptake of family planning was low; but this has changed significantly with family planning now an item of demand.   Overall, this is an extraordinary study and I learned a lot. Thank you.

# Reviewer report: Reviewer response for version 1

**DOI:** 10.21956/wellcomeopenres.28177.r144720

**Published:** 2026-01-29

**Authors:** Aula Abbara

**Affiliations:** 1 Imperial College, london, UK; 2 Infectious Diseases, Imperial College London Faculty of Medicine (Ringgold ID: 4957), London, England, UK

Many thanks for the opportunity to review this report.

 This article summarises the founding and evolution of a unique and remarkable project which was established 40 years ago along the Thai-Burma border. It provides key insights from data on 94,645 pregnancies and the impacts of malaria and other conditions e.g. NCDs, neurodevelopment, nutrition among a population of refugees/ migrants. Beyond this, research and evidence developed - based on this cohort - has changed WHO malaria treatment guidance in pregnancy and supported the development of relevant tools. This report also highlights why such research/ initiatives are important: low mean height and other demographic differences between this population and European/ American populations emphasise why particular population cohorts can provide important adaptations to international guidance which may be based on European/ American populations

 The description and presentation is clear throughout and is highly compelling - capturing the initial needs in what was at that time a highly volatile setting through to the involvement of MSF and evolution into SMRU. It captures useful contextual information and how this impacts the population, its mobility and particular needs including around pregnancy, maternal and neonatal health and malaria. It describes how the response to the population has evolved over time based on changing needs. In particular, it speaks to the changing nutritional status, including in high BMI over time and what this means in terms of monitoring gestational weight gain but also how health workers need to respond to address health literacy.

 The report also highlights some of the challenges in collating and maintaining data for such a cohort over the almost 40 years since it was established - alongside the transition from paper based records through to digitalisation and how this was approached.

 There is very little to critique in this report. The authors provide information about data availability and potential for data requests and collaboration. It also usefully highlights the numerous academic outputs from SMRU, grouping them in topics of interest.

 It is not the main am of the report which is why it may not be included in detail, however, in figure 3 and its descriptions, it would be interesting to know 1. what the authors think has stalled maternal mortality and 2. any more information about trends of malaria mortality to be highlighted (though aware some of the authors' existing academic publications capture this, it is challenging in a mobile population and the authors acknowledge the more than 100 women who passed away.) Lastly, a short conclusion after 'What are the main strengths and weaknesses of the study?' would be useful for the reader to provide the key messages the authors would like reader to take away. 

 This report describes a remarkable and unique project which has had significant impacts on the understanding of malaria in pregnancy, related comorbidities and has changed practice. It captures its evolution, key time points and highlights the impacts and outputs from this work.

Is the study design appropriate for the research question?

Yes

Is the rationale for, and objectives of, the study clearly described?

Yes

Are sufficient details of the methods provided to allow replication by others?

Yes

Are the datasets clearly presented in a useable and accessible format?

No

Reviewer Expertise:

Infectious diseases, malaria, AMR, conflict

I confirm that I have read this submission and believe that I have an appropriate level of expertise to confirm that it is of an acceptable scientific standard.

# Response

**Published:** 2026-03-01

**Authors:** Rose McGready

**Affiliations:** Shoklo Malaria Research Unit, Thailand

Many thanks to Reviewer 1 for their thoughtful review and highly useful suggestions. In response to the comments we have amended the manuscript. A point by point response follows: It is not the main aim of the report which is why it may not be included in detail, however, in figure 3 and its descriptions, it would be interesting to know 1. what the authors think has stalled maternal mortality and We are analysing and writing an individual case analysis for maternal mortality. **The main aim of figure 3 was to highlight the overwhelming efficiency of *P. falciparum *as a single lethal agent in non/pauci-immune women. **2. any more information about trends of malaria mortality to be highlighted (though aware some of the authors' existing academic publications capture this, it is challenging in a mobile population, and the authors acknowledge the more than 100 women who passed away.) A **s above and thank you for point this out – we have added text to better emphasize this**. Lastly, a short conclusion after 'What are the main strengths and weaknesses of the study?' would be useful for the reader to provide the key messages the authors would like reader to take away. **Thank you – added.**

 This report describes a remarkable and unique project which has had significant impacts on the understanding of malaria in pregnancy, related comorbidities and has changed practice. It captures its evolution, key time points and highlights the impacts and outputs from this work. Thank you **Is the rationale for, and objectives of, the study clearly described?** Yes **Is the study design appropriate for the research question?** Yes **Are sufficient details of the methods provided to allow replication by others?** Yes **Are the datasets clearly presented in a useable and accessible format?** No We checked the links in the online version and they seen ok.
